# Grape-Seed Proanthocyanidins Modulate Adipose Tissue Adaptations to Obesity in a Photoperiod-Dependent Manner in Fischer 344 Rats

**DOI:** 10.3390/nu15041037

**Published:** 2023-02-19

**Authors:** Èlia Navarro-Masip, Marina Colom-Pellicer, Francesca Manocchio, Anna Arola-Arnal, Francisca Isabel Bravo, Begoña Muguerza, Gerard Aragonès

**Affiliations:** Nutrigenomics Research Group, Department of Biochemistry and Biotechnology, Universitat Rovira i Virgili, Marcel·lí Domingo 1, 43007 Tarragona, Spain

**Keywords:** adiponectin, BAT, flavonoids, GSPE, seasonal rhythms

## Abstract

Seasonal rhythms drive metabolic adaptations that influence body weight and adiposity. Adipose tissue is a key regulator of energy homeostasis in the organism, and its healthiness is needed to prevent the major consequences of overweight and obesity. In this context, supplementation with proanthocyanidins has been postulated as a potential strategy to prevent the alterations caused by obesity. Moreover, the effects of these (poly)phenols on metabolism are photoperiod dependent. In order to describe the impact of grape-seed proanthocyanidins extract (GSPE) on important markers of adipose tissue functionality under an obesogenic environment, we exposed Fischer 344 rats to three different photoperiods and fed them a cafeteria diet for five weeks. Afterwards, we supplemented them with 25 mg GSPE/kg/day for four weeks. Our results revealed that GSPE supplementation prevented excessive body weight gain under a long photoperiod, which could be explained by increased lipolysis in the adipose tissue. Moreover, cholesterol and non-esterified fatty acids (NEFAs) serum concentrations were restored by GSPE under standard photoperiod. GSPE consumption slightly helped combat the obesity-induced hypertrophy in adipocytes, and adiponectin mRNA levels were upregulated under all photoperiods. Overall, the administration of GSPE helped reduce the impact of obesity in the adipose tissue, depending on the photoperiod at which GSPE was consumed and on the type of adipose depots.

## 1. Introduction

Obesity is a public health issue worldwide that has nearly tripled since 1975, and in 2016, 39% of adults were overweight and 13% were obese [1]. The principal cause of obesity is an energy imbalance between food intake and energy expenditure [2,3,4]. The World Health Organization (WHO) and other entities report that obesity is preventable by consuming healthier foods, such as vegetables, legumes, whole grains, and nuts, and increasing physical activity by up to 150 min per week for adults. Moreover, seasonal changes affect the metabolic features that control energy balance, such as food intake, adiposity, or energy metabolism [5,6]. In this sense, humans have a higher biological susceptibility to gaining weight in the summer than in the winter, meaning that adopting unhealthy habits in the summer can contribute to weight gain and obesity. Indeed, children gained more weight in the summer holidays rather than in the Christmas holidays [7,8]. Despite the recommendations, the incidence of obesity is still increasing every year; therefore, the need for the development of new strategies to ameliorate obesity and obesity-related disorders becomes greater.

A healthy functionality of the white adipose tissue (WAT) is crucial to prevent metabolic disorders since alterations in WAT metabolism can lead to an inflammatory response and systemic complications [9]. In an obesogenic environment, the healthiness of WAT is seriously threatened, and the general consequences that can arise from this situation depend on WAT location. In fact, the WAT is largely distributed throughout the organism, being classified into subcutaneous WAT and visceral WAT [10]. The subcutaneous WAT (sWAT) has a better adaptation to an obesogenic environment, yet it is capable of better modulating the inflammatory response and preventing lipotoxicity. However, the visceral WAT (vWAT) is less tolerant to a calory-excess situation: its development under obesity has a stronger impact on systemic inflammation, as it produces a considerable amount of pro-inflammatory cytokines and promotes an exacerbated immune response, followed by lipotoxicity [11].

The brown adipose tissue (BAT) appears as an important thermogenic tissue in mammals that strongly contributes to energy metabolism. Its role and characteristics are different from WAT: briefly, brown adipocytes contain high amounts of mitochondria and low amounts of small lipid droplets [12]. Moreover, they count on the presence of Uncoupling Protein 1 (UCP1) in the membrane of their mitochondria, which is a protein capable of dissipating the electron respiratory chain and using the energy to produce heat [12]. Consequently, its adaptation to distinct environments such as obesity or seasonality is different from the WAT and can help the organism face metabolic complications [6].

The consumption of specific bioactive compounds, such as (poly)phenols, is highly associated with health benefits [13]. These molecules have been related to a reduced incidence of metabolic disorders such as obesity, glucose intolerance, or cardiovascular disease [14,15]. In addition, phenolic compounds have shown synchronizing characteristics that can ease the metabolic adaptations of the organism to its environment [16,17]. In fact, an influence of photoperiod on the metabolic effects of grape-seed proanthocyanidin extract (GSPE) was recently demonstrated in healthy and obese animals. More specifically, GSPE modulated hepatic glucose and lipid metabolism in a photoperiod-dependent manner [18]. Moreover, GSPE effects on microbiota were dependent on photoperiod in obese animals [19]. GSPE contributed to the adaptation to new photoperiods by regulating body weight and energy expenditure [20]. However, the seasonal effects of GSPE consumption on adipose tissue adaptations to obesity have not been reported to date. Therefore, the aim of this study was to explore the effects of GSPE seasonal consumption on the functionality of different adipose tissue depots (BAT, visceral, and subcutaneous WAT) in animals fed a cafeteria (CAF) diet.

## 2. Materials and Methods

### 2.1. Grape-Seed Proanthocyanidin Extract

GSPE was kindly provided by Les Dérivés Résiniques et Terpéniques (Dax, France). According to the manufacturer, the GSPE composition used in this study contained monomers (21.3%), dimers (17.4%), trimers (16.3%), tetramers (13.3%), and oligomers (5–13 units; 31.7%) of proanthocyanidins. The exact phenolic composition of GSPE was determined by HPLC-MS/MS (TOF 6210, Agilent) [21], according to what was described by Quiñones et al. [22], and can be found in Supplementary Table S1.

### 2.2. Animal Experimental Procedure

Eight-week-old male Fischer 344 rats (*n* = 72) were purchased from Charles River Laboratories (Barcelona, Spain) and pair-housed in animal quarters at 22 °C with a light/dark period of 12 h. Animals had an adaptation period of 1 week fed with standard diets (STD) (Panlab, Barcelona, Spain) and tap water ad libitum. After the adaptation period, animals were submitted to three different light schedules for 9 weeks to mimic seasonal day lengths: L12 photoperiod (12 h light–12 h darkness); L18 photoperiod (18 h light–6 h darkness); and L6 photoperiod (6 h light–18 h darkness). Animals in each photoperiod group were further divided into three more groups (*n* = 8); one of them was fed an STD diet *ad libitum,* and the other two were fed cafeteria (CAF) diet *ad libitum.* The CAF diet consisted of biscuits with pâté, biscuits with cheese, *ensaïmada* (sweetened pastry), bacon, carrots, and sweetened milk (20% sucrose *w/v*) in addition to the standard diet (STD Panlab A04, Panlab, Barcelona, Spain). The CAF diet is a highly palatable diet that is able to induce voluntary hyperphagia. Its composition was 10% protein, 31.9% fat, and 58.1% carbohydrates. Each component of the CAF diet was freshly provided to the animals daily, and they could choose and eat ad libitum. At week 6, the 4-week treatment period started while the animals kept eating the same type of diet. The three STD diet groups received the vehicle (VH) treatment, consisting of an oral dose of water and sweetened milk. Three of the CAF groups (one from each photoperiod) received VH, and the other three received 25 mg/kg/day of GSPE diluted in water and sweetened milk. At the end of the experiment, animals were fasted for 3 h and then sacrificed by live decapitation. Total blood was collected from the neck and then centrifuged (1500× *g*, 15 min, 4 ºC) to obtain serum. All adipose tissue depots were excised, weighted and immediately frozen into liquid nitrogen, one piece of epididymal white adipose tissue (eWAT) and inguinal white adipose tissue (iWAT) were also kept in formol for histological analysis. Both serum and tissues were stored at −80 °C until further use. A schematic representation of the experimental design can be found in Supplementary Figure S1. The Ethics Review Committee for Animal Experimentation of the University Rovira i Virgili (Tarragona, Spain) and the Generalitat de Catalunya approved all the procedures of the investigation (reference number 9495), which was carried out in accordance with the ethical standards and the Declaration of Helsinki.

### 2.3. Serum Analysis

Serum glucose, total cholesterol, and triglycerides (TG) were measured with enzymatic colorimetric kits (QCA, Barcelona, Spain). Serum non-esterified fatty acids (NEFAs) were analyzed with the enzymatic colorimetric HR NEFA series kit (Wako, CA, USA).

### 2.4. Histology of Adipose Tissues

Frozen iWAT and eWAT samples were thawed and fixed in 4% formaldehyde. Tissues underwent successive dehydration and paraffin infiltration immersion (Citadel 2000, HistoStar, Thermo Scientific, Madrid, Spain) and the paraffin blocks were cut into 2-μm-thick sections using a microtome (Microm HM 355S, ThermoScientific). The sections were subjected to automated hematoxylin–eosin staining (Varistain Gemini, Shandom, Thermo Scientific). Sections were observed and acquired at ×10 magnification using AxioVision ZeissImaging software (Carl Zeiss Iberia, S.L., Madrid, Spain). The area of adipocytes was measured using the Adiposoft open-source software (CIMA, University of Navarra, Pamplona, Spain). Four fields per sample were measured, and six samples per group were analyzed. The adipocyte area was calculated from the average value of the area of cells in all measured fields for each sample. The total adipocyte volume was calculated using the formula $\left[\frac{\pi }{6}\right]\times \left[3\sigma^{2}\times \bar{d}+{\bar{d}}^{3}\right]$, where $\bar{d}$ is the mean diameter and σ is the standard deviation of the diameter. Afterwards, this value was converted to the adipocyte weight using the adipocyte density (0.92 g/mL). In order to obtain the total adipocyte number in each depot, the weights of iWAT and eWAT depots were divided by the adipocyte weight. The frequencies of adipocytes were calculated by dividing all counted cells per sample into two groups according to their area, <3000 μm^2^ or >3000 μm^2^; then, the total number of counted adipocytes to calculate the percentage of adipocytes in both categories.

### 2.5. Gene Expression Analysis

Total RNA from iWAT, eWAT and BAT was extracted using TRIzol reagent (Thermo Fisher Scientific, Barcelona, Spain) following the manufacturer’s protocol. RNA was quantified in NanoDrop ND-1000 spectrophotometer (Thermo Scientific, Wilmington, DE, USA). The integrity of the RNA was evaluated by the RNA integrity number (RIN) trough 2100 Bioanalyzer Instrument (Agilent Technologies). A RIN higher than six was accepted for total RNA samples. cDNA was synthesized using the High-Capacity cDNA Reverse Transcription Kit (Applied Biosystems, Barcelona, Spain) in a Multigene ThermalCycler (Labnet, Madrid, Spain). The cDNA was subjected to a quantitative reverse transcriptase polymerase chain reaction amplification using iTaq™Universal SYBR Green Supermix (Bio-Rad, Madrid, Spain) in the 7900HT Fast Real-Time PCR System (Applied Biosystems). The primers used for the different genes are described in Supplementary Table S2 and were obtained from Biomers.net (Ulm, Germany). The relative expression of each gene was calculated according to *cyclophilin peptidylprolyl isomerase A* (*Ppia*) mRNA levels and normalized to the levels measured in the corresponding control group. The ∆∆Ct method was used and corrected for primer efficiency [23].

### 2.6. Statistical Analysis

The effects of both CAF diet administration and seasonal GSPE supplementation on biometric, biochemical, and serum variables as well as gene expression were evaluated by a two-way ANOVA with Tukey’s *post hoc* test for multiple comparisons. In addition, for biometric, biochemical, and serum variables, a one-way ANOVA followed by Tukey’s *post hoc* test was conducted to detect significant differences between groups in the same photoperiod. GraphPad Prism 9 (GraphPad Software, La Jolla, CA, USA) was used for all statistical analysis. The values are expressed as means ± SEM. *p*< 0.05 was considered significant.

## 3. Results

### 3.1. GSPE Consumption Restored Cholesterol and NEFAs Serum Concentrations in a Photoperiod-Dependent Manner in Obese Animals

After nine weeks of study, animals fed the CAF diet significantly increased their body weight, body weight gain, and food intake (Table 1). However, the four-week GSPE supplementation was able to significantly reduce body weight gain and food intake in all groups. When we conducted one-way ANOVA tests to assess the individual effects of GSPE on each photoperiod, we detected that the greater effects on reducing body weight gain were in animals exposed to L18. Furthermore, the CAF diet significantly increased total fat mass and the mass of individual adipose tissue depots (iWAT, eWAT, and BAT) in all animals. In this case, GSPE supplementation did not significantly affect these values.

A diet effect was also observed in all circulating metabolic parameters, where CAF consumption significantly increased serum concentrations of glucose, cholesterol, TG, and NEFAs. However, GSPE supplementation was able to restore cholesterol and NEFAs levels in a photoperiod-dependent manner.

### 3.2. GSPE Consumption Restored Adipocyte Morphology in iWAT in a Photoperiod-Independent Manner

**Figure 1:**
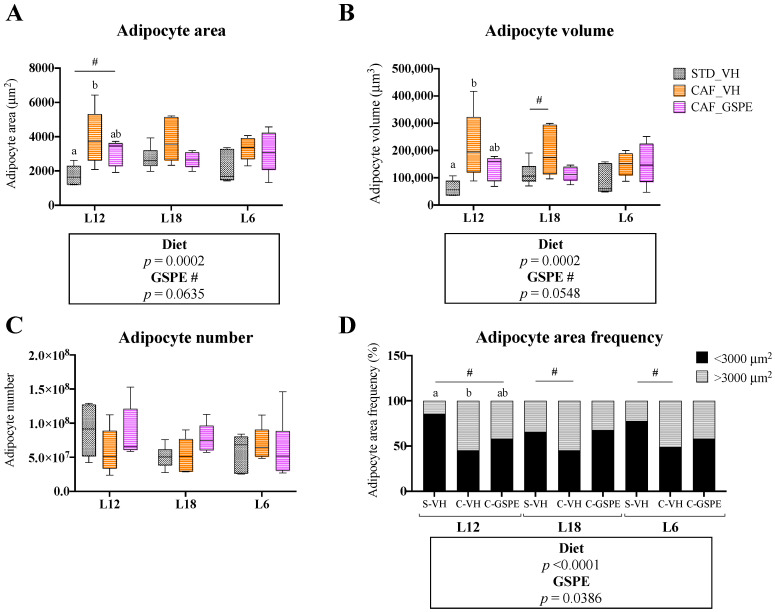
Adipocyte area (**A**), adipocyte volume (**B**), adipocyte number (**C**) and adipocyte area frequencies (**D**) of inguinal white adipose tissue (iWAT) of rats treated with grape seed proanthocyanidin extract (GSPE) at different photoperiods, 12 h of light:12 h of darkness (L12); 18 h of light:6 h of darkness (L18); 6 h of light:18 h of darkness (L6). Histological study of iWAT morphology of Fisher 344 rats fed with standard diet (STD) or cafeteria diet (CAF) for 9 weeks and treated with GSPE (25 mg/kg body weight) or vehicle (VH) the last 4 weeks of the study; STD-VH group; CAF-VH group; CAF-GSPE group, under different photoperiods; L12, L18 or L6. For adipocyte area frequency, adipocytes were distributed in two groups according to their areas (<3000 or >3000 μm^2^). The results are presented as the mean ± SEM (*n* = 6). Significant differences were assessed through two-way ANOVA analysis (*p* < 0.05). Diet: diet effect within VH groups; GSPE: GSPE consumption effect within CAF groups; **#**: tendency (0.05 < *p* < 1). Different letters denote significant differences within each photoperiod group (assessed with one-way ANOVA followed by Tukey’s *post hoc* test, *p* < 0.05; **#**: tendency: 0.05 < *p* < 1).

The histological analysis of iWAT showed a significant effect of the CAF diet on both area and volume of adipocytes in iWAT, which were increased in obese animals (Figure 1A,B, Supplementary Figure S2). Consequently, the adipocyte area distribution also changed, and the number of larger adipocytes increased with respect to the smaller ones (Figure 1D). However, no differences were detected in adipocyte number (Figure 1C), and, when we applied one-way ANOVA analyses to assess the significant effects within each photoperiod, we only observed statistical significance on L12 animals. Noteworthy, as indicated by two-way ANOVA analyses, GSPE consumption reduced the CAF diet impact because the cellular profile of CAF-fed animals supplemented with GSPE was more similar to the STD diet fed groups, showing reduced adipocyte area and volume. No interaction between GSPE and photoperiod was detected, but the GSPE effect only reached significance in L12 groups after applying one-way ANOVA tests.

### 3.3. Gene Expression of iWAT Was Affected by GSPE in a Photoperiod-Dependent Manner

**Figure 2:**
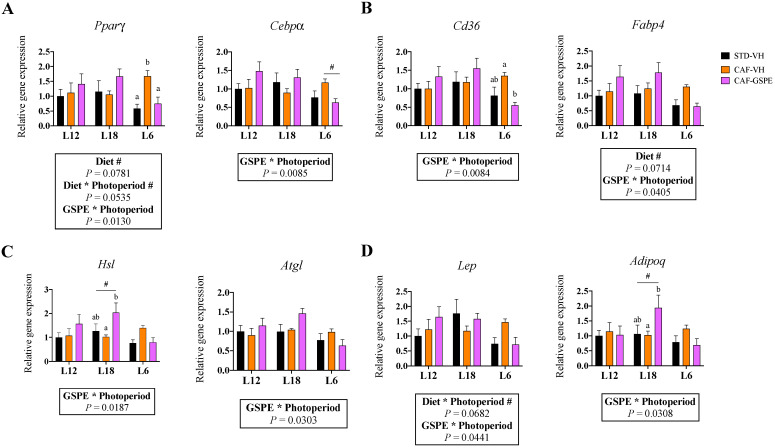
Effect of grape seed proanthocyanidin extract (GSPE) consumption at different photoperiods, 12 h of light:12 h of darkness (L12); 18 h of light:6 h of darkness (L18); 6 h of light:18 h of darkness (L6) on the expression of gens related to adipogenesis, lipogenesis, lipolysis and adipokines in inguinal white adipose tissue (iWAT) of diet-induced obese rats. Fisher 344 rats were fed standard diet (STD) or cafeteria diet (CAF) for 9 weeks and treated with GSPE (25 mg/kg body weight) or vehicle (VH) the last 4 weeks of the study; STD-VH group; CAF-VH group; CAF-GSPE group, under different photoperiods; L12, L18 or L6. The expression of genes involved in adipogenesis (**A**), lipogenesis (**B**), lipolysis (**C**) and adipokines (**D**) was measured by qPCR and normalized by *Ppia*. The relative expression (presented as fold-change) of CAF-VH and CAF-GSPE was normalized to the corresponding STD-VH control group (L12 photoperiod). Significant differences were assessed through two-way ANOVA analysis (*p* < 0.05). Diet: diet effect within VH groups; Diet * Photoperiod: interaction effect between diet and photoperiod within VH groups; GSPE * Photoperiod: interaction effect between GSPE consumption and photoperiod within CAF groups. Different letters denote significant differences within each photoperiod group (assessed with two-way ANOVA followed by Tukey’s *post hoc* test, *p* < 0.05; **#**: tendency: 0.05 < *p* < 1).

However, after exploring the expression of the key metabolic genes in iWAT, we detected an interaction effect between GSPE and photoperiod in the expression levels of the adipogenic genes *Cebpα* and *Pparγ* (Figure 2A). The effect was only observed in animals exposed to the L6 photoperiod, in which the expression of these genes was significantly restored in response to GSPE consumption. A similar effect was detected in the lipid transport-related genes *Cd36* and *Fabp4* (Figure 2B), the lipolysis-related genes *Hsl* and *Atgl* (Figure 2C), and the adipokine genes *Lep* and *Adipoq* (Figure 2D). Differently, no significant effects in response to GSPE consumption were detected in the gene expression of the lipogenic genes *Acacα* and *Fasn* (Supplementary Figure S3).

### 3.4. GSPE Consumption Restored Adipocyte Morphology in eWAT in a Photoperiod-Independent Manner

**Figure 3:**
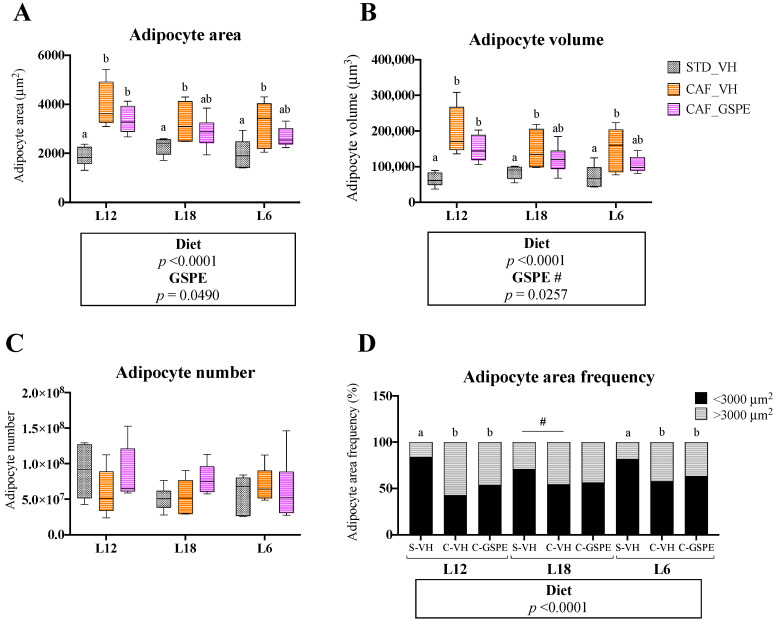
Adipocyte area (**A**), adipocyte volume (**B**), adipocyte number (**C**) and adipocyte area frequencies (**D**) of epididymal white adipose tissue (eWAT) of rats treated with grape seed proanthocyanidin extract (GSPE) at different photoperiods, 12 h of light:12 h of darkness (L12); 18 h of light:6 h of darkness (L18); 6 h of light:18 h of darkness (L6). Histological study of eWAT morphology of Fisher 344 rats fed with standard diet (STD) or cafeteria diet (CAF) for 9 weeks and treated with GSPE (25 mg/kg body weight) or vehicle (VH) the last 4 weeks of the study; STD-VH group; CAF-VH group; CAF-GSPE group, under different photoperiods; L12, L18 or L6. For adipocyte area frequency, adipocytes were distributed in two groups according to their areas (<3000 or >3000 μm^2^). The results are presented as the mean ± SEM (*n* = 6). Significant differences were assessed through two-way ANOVA analysis (*p* < 0.05). Diet: diet effect within VH groups; GSPE: GSPE consumption effect within CAF groups; #: tendency (0.05 < *p* < 1). Different letters denote significant differences within each photoperiod group (assessed with one-way ANOVA followed by Tukey’s *post hoc* test, *p* < 0.05; **#**: tendency: 0.05 < *p* < 1).

Similar to iWAT, a significant effect of both the CAF diet and GSPE consumption was detected in the histological analysis of eWAT. Again, the CAF diet increased adipocyte area and volume, whereas GSPE consumption attenuated this significant increase regardless of the photoperiod (Figure 3A,B, Supplementary Figure S4). However, these changes in response to proanthocyanidins were not fully reflected by adipocyte number or adipocyte area frequency (Figure 3C,D), and one-way ANOVA tests did not show significancy between CAF-VH and CAF-GSPE groups on area and volume values under any photoperiod.

### 3.5. GSPE Consumption Influenced the Expression of Adipogenic Genes in eWAT in a Photoperiod-Dependent Manner

**Figure 4:**
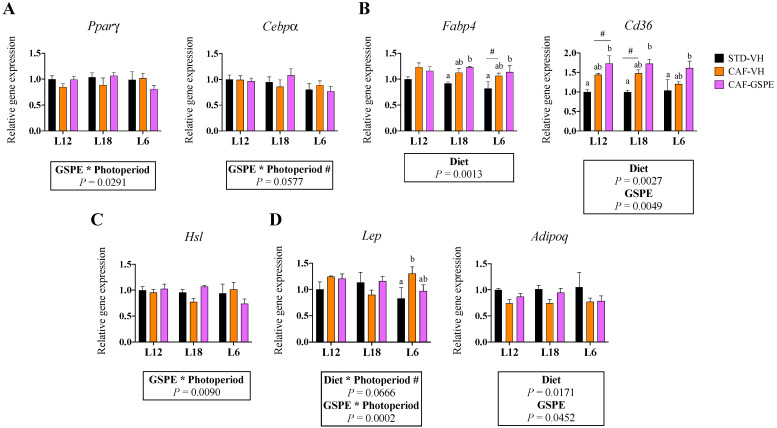
Effect of grape seed proanthocyanidin extract (GSPE) consumption at different photoperiods, 12 h of light:12 h of darkness (L12); 18 h of light:6 h of darkness (L18); 6 h of light:18 h of darkness (L6) on the expression of gens related to adipogenesis, lipid transport, lipolysis and adipokines in epididymal white adipose tissue (eWAT) of diet-induced obese rats. Fisher 344 rats were fed standard diet (STD) or cafeteria diet (CAF) for 9 weeks and treated with GSPE (25 mg/kg body weight) or vehicle (VH) the last 4 weeks of the study; STD-VH group; CAF-VH group; CAF-GSPE group, under different photoperiods; L12, L18 or L6. The expression of genes involved in adipogenesis (**A**), lipid transport (**B**), lipolysis (**C**) and adipokines (**D**) was measured by qPCR and normalized by *Ppia*. The relative expression (presented as fold-change) of CAF-VH and CAF-GSPE was normalized to the corresponding STD-VH control group (L12 photoperiod). Significant differences were assessed through two-way ANOVA analysis (*p* < 0.05). Diet: diet effect within VH groups; GSPE: GSPE consumption effect within CAF groups; Diet * Photoperiod: interaction effect between diet and photoperiod within VH groups; GSPE * Photoperiod: interaction effect between GSPE consumption and photoperiod within CAF groups; #: tendency: 0.05 < *p* < 1. Different letters denote significant differences within each photoperiod group (assessed with two-way ANOVA followed by Tukey’s *post hoc* test, *p* < 0.05; **#**: tendency: 0.05 < *p* < 1).

As observed in iWAT, when we explored the gene expression profile of metabolic genes in eWAT, an interaction between GSPE and photoperiod was significantly detected in adipogenic genes (Figure 4A). Moreover, GSPE consumption significantly upregulated the gene expression of *Fabp4* (in L18 and L6 animals) and of *Cd36* (Figure 4B). Similarly, an interaction effect between GSPE consumption and photoperiod was observed in the lipolytic gene *Hsl* (Figure 4C). As expected, the CAF diet significantly downregulated the expression of the lipogenic genes *Acacα* and *Fasn* in all animal groups (Supplementary Figure S5).

Interestingly, an interaction effect between GSPE and photoperiod was also detected in the expression of *Lep*, which was reduced in response to GSPE consumption in the L12 and L6 groups but significantly upregulated in the L18 animals (Figure 4D). In addition, *Adipoq* gene expression was reduced by the CAF diet in all groups, but a tendency for GSPE to reverse this decrease was observed in animals subjected to the L12 and L18 photoperiods. Finally, a significant effect of the CAF diet on the expression of the inflammatory genes *Il6* and *Tnfα* was also observed (Supplementary Figure S5).

### 3.6. GSPE Consumption Reverted the Obesity-Induced Downregulation of Pgc1α in BAT in a Photoperiod-Dependent Manner

**Figure 5:**
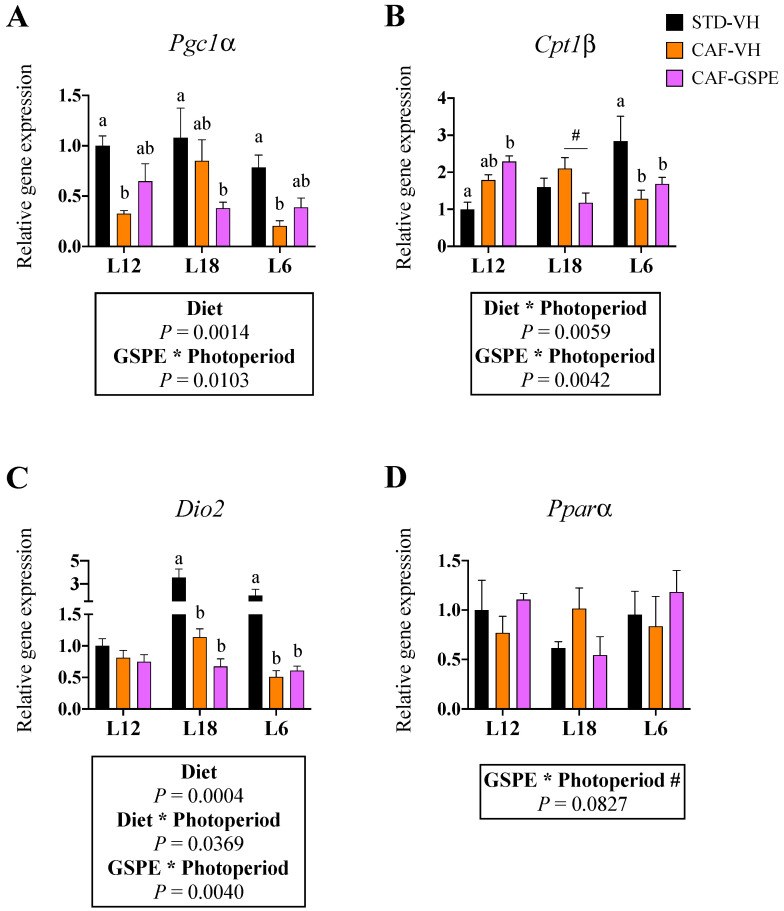
Effect of grape seed proanthocyanidin extract (GSPE) consumption at different photoperiods, 12 h of light:12 h of darkness (L12); 18 h of light:6 h of darkness (L18); 6 h of light:18 h of darkness (L6) on the expression of key metabolic genes in brown adipose tissue (BAT) of diet-induced obese rats. Fisher 344 rats were fed standard diet (STD) or cafeteria diet (CAF) for 9 weeks and treated with GSPE (25 mg/kg body weight) or vehicle (VH) the last 4 weeks of the study; STD-VH group; CAF-VH group; CAF-GSPE group, under different photoperiods; L12, L18 or L6. The gene expression of *Pgc1α* (**A**), *Cpt1β* (**B**), *Dio2* (**C**) and *Pparα* (**D**) was measured by qPCR and normalized by *Ppia*. The relative expression (presented as fold-change) of CAF-VH and CAF-GSPE was normalized to the corresponding STD-VH control group (L12 photoperiod). Significant differences were assessed through two-way ANOVA analysis (*p* < 0.05). Diet: diet effect within VH groups; Diet * Photoperiod: interaction effect between diet and photoperiod within VH groups; GSPE * Photoperiod: interaction effect between GSPE consumption and photoperiod within CAF groups; #: tendency: 0.05 < *p* < 1. Different letters denote significant differences within each photoperiod group (assessed with two-way ANOVA followed by Tukey’s post hoc test, *p* < 0.05; #: tendency: 0.05 < *p* < 1).

Being BAT such an important tissue in energy homeostasis, especially in rodents, we analyzed the most important components that drive the main metabolic pathways of brown adipocytes’ activity. Despite the fact that no significant effects of CAF diet or GSPE administration on the gene expression of *Ucp1* were detected (Supplementary Figure S6), an interaction effect between GSPE and photoperiod was observed in the gene expression of *Pgc1α*, *Cpt1β,* and *Dio2* (Figure 5A–C). Particularly, GSPE supplementation subtly upregulated the expression of these genes under L12 and L6 photoperiods, while it downregulated its expression under L18. Moreover, the CAF diet downregulated the expression of *Pgc1α* in all photoperiods and the expression of *Dio2* in the L18 and L6 groups. Finally, mRNA levels of *Cpt1β* were influenced by the CAF diet in a photoperiod-dependent manner.

Moreover, *Cd36*, *Lpl, Pparγ,* and *Prdm16* gene expression was significantly downregulated by the CAF diet in all animal groups (Supplementary Figure S6). However, an interaction effect between GSPE and photoperiod was observed in the gene expression of *Pparα*, which tended to be upregulated in the L12 and L6 groups but not in the L18 animals (Figure 5D).

## 4. Discussion

The beneficial effects of (poly)phenols on health and their natural presence in vegetable foods make these molecules of high interest in research. Additionally, a (poly)phenol-rich diet or the development of natural-based extracts as an alternative for preventing or treating metabolic disorders is more likely to be accepted by society than synthetic options [24]. In this context, (poly)phenol consumption can help reduce the negative effects of a disrupted metabolism in the adipose tissue and, consequently, in the whole organism [25]. Moreover, changes in the atmosphere can drive metabolic adaptations in mammals [26], meaning that seasonal rhythms can impact the effects of (poly)phenol consumption in adipose tissue. However, these effects have not been reported yet, even if evidence exists about the impact of seasonal consumption of (poly)phenols in other metabolic tissues such as the liver or the gut microbiota [18,19]. Our study aimed to elucidate the effects of proanthocyanidin seasonal consumption on the adaptations of the adipose tissue to an obesogenic environment. For this purpose, Fischer 344 rats were exposed to different photoperiod schedules in order to mimic the seasons of the year. Then, animals were fed a CAF diet and supplemented with either VH or 25 mg GSPE/ kg/day for four weeks. The dose of GSPE was chosen based on previous studies that showed metabolic effects with the same dose [27].

After nine weeks of study, body weight gain was reduced in response to proanthocyanidin consumption in CAF-fed animals. These results were particularly marked when GSPE was consumed at L18 than when it was at L12 and L6 photoperiods. According to the seasonal adaptations to photoperiod, increased weight gain in long photoperiods appears as a natural metabolic modulation in mammals, which, contrarily, lose more weight in short photoperiods [28]. Nevertheless, GSPE consumption resulted in a significant reduction in food intake only in animals subjected to L12 and L6 photoperiods but not in L18 animals. The effects of proanthocyanidins on reducing food intake were previously reported in Wistar rats [29] and were attributed to the astringency of these (poly)phenols [30]. Other mechanisms that could explain the GSPE-derived inhibition of food intake involve the central nervous system. On the one hand, enhanced leptin signaling was detected in obese rats supplemented with GSPE [31]. Furthermore, proanthocyanidins were shown to induce glucagon-like peptide (GLP-1) secretion, which is capable of increasing satiety and inhibiting feeding [32]. Nevertheless, considering the strong seasonal impact of long photoperiods on feeding behavior, which remains unaltered even with increased leptin levels, reflecting reduced leptin sensitivity under summer-like conditions, it would be possible that the GSPE lowering effects on food intake were not adopted by animals under the L18 photoperiod [33,34,35]. Similarly, even if previous studies observed increased energy expenditure in animals supplemented with GSPE under standard photoperiods [29,36], a previous study from our group showed that the energy expenditure of CAF-fed rats exposed to L18 and supplemented with GSPE for one week tended to be reduced [20], which suggests that the observed decrease in body weight gain in L18-CAF animals supplemented with proanthocyanidins in our study should be explained by other mechanisms. For instance, it was recently reported that obese rats exposed to L18 showed greater changes in the gut microbiota composition in response to proanthocyanidin consumption compared to animals exposed to L12 and L6 photoperiods. Particularly, GSPE consumption under L18 resulted in increased levels of *Bifidobacterium* and *Coprobacillus*, while decreased *Klebsiella* to levels associated with reduced body weight gain and obesity [19]. Moreover, other studies indicated that proanthocyanidins can modulate nutrient absorption in the gastrointestinal tract, through inhibiting digestive enzymes such as α-Amylase and α-Glucosidase, resulting in a 20% decrease in the amount of absorbed energy [29,37]. In future studies, it would be interesting to calculate energy/food efficiency in these animals in order to understand the proanthocyanidin-induced mechanism driving the reduction in body weight gain under the L18 photoperiod.

Given that WAT functionality responds to changes in photoperiod and that obesity drives major changes to the structure and activity of this tissue, histological analyses of WAT depots were performed. As expected, we observed a strong impact of the CAF diet on the area and volume of adipocytes, which were larger compared to animals fed a chow diet. These changes are common in obesity and contribute to the altered functionality of the WAT. In fact, under an obesogenic environment, the WAT needs to store the excess calories, which can be done through two processes: hyperplasia or hypertrophy [38]. The hyperplasia expansion is considered the healthy expansion, where the number of adipocytes increases via adipogenesis and they maintain their functionality and insulin sensitivity [9,38]. However, when hypertrophy occurs, existing adipocytes need to incorporate the excess of fat, increasing their volume and disrupting their functionality [9]. These dysfunctional adipocytes are less glucose tolerant and become pro-inflammatory, producing cytokines such as IL-6 or TNFα, which can induce macrophage infiltration in the adipose tissue and provoke a systemic immune response [39]. Adipose tissue expansion partly depends on the adipose depot; indeed, under healthy conditions, hyperplasia is more commonly developed in subcutaneous depots, while visceral depots are more susceptible to hypertrophy [40]. Additionally, it has been studied that hypertrophic visceral adipose tissue has a stronger negative impact on the whole metabolism of the organism, being highly associated with the metabolic syndrome [39]. Therefore, it is important to maintain the integrity and healthiness of this adipose depot. Furthermore, in our study, proanthocyanidin consumption reversed the effect induced by the CAF diet in iWAT regardless of the photoperiod, as adipocyte areas and volumes were at similar levels to their healthy counterparts in all photoperiod conditions. Similar results have been reported with GSPE supplementation in iWAT [25]. Further, the administration of blueberry polyphenol extract resulted in a reduced adipocyte area in the eWAT [41]. Nonetheless, in our study, this effect was less clear in eWAT, where a significant effect of GSPE was observed but the individual differences within groups in each photoperiod were not significant. In addition, when we studied the gene expression of inflammatory markers in eWAT (*Il6* and *Tnfα*), no effects were observed in response to GSPE consumption.

One of the main roles of WAT is to regulate energy balance in the organism. Therefore, lipid turnover is constantly active, involving distinct metabolic pathways that control fat storage and release from the WAT. The process that drives the formation of new adipocytes is named adipogenesis and is mainly driven by the master adipogenic molecules PPARγ and C/EBPs [42]. In our study, gene expression of *Pparγ* and *Cebpα* in both iWAT and eWAT showed an interaction effect between GSPE administration and photoperiod. In iWAT, we observed that GSPE increased its gene expression only in the L12 and L18 groups. Similar effects were previously observed in response to cyanidin-3-glucoside administration [43] and could explain the differences observed in the histological analyses, where the presence of larger adipocytes provoked by the CAF diet was partly reversed in response to GSPE consumption in L12 and L18 animals. Hence, increased adipogenesis would favor hyperplasia expansion rather than hypertrophy. Furthermore, the lipid transport-related genes (*Fabp4* and *Cd36*) were also affected by GSPE consumption in iWAT (L12 and L18 groups) and in eWAT (all groups), which could suggest a beneficial role for proanthocyanidins in helping release circulating TG. In fact, animals consuming GSPE at L12 and L18 photoperiods showed reduced serum TG concentrations compared to VH groups. However, another interaction effect between GSPE and photoperiod on the expression of lipolysis-related genes was detected in both iWAT and eWAT, which were upregulated in response to GSPE consumption only in the L12 and L18 groups. This activation of lipolysis could be involved in reducing the size of existing adipocytes, contributing to β-oxidation and global energy homeostasis. Moreover, the reduction in body weight gain observed in L18 animals could be partly explained by the increased lipolysis detected in these animals. In fact, supplementation with blueberry (poly)phenols suggested increased lipolysis in the WAT [41], and the lipolytic effects of other types of (poly)phenols such as epigallocatechin-gallate (EGCG) or resveratrol are already established [44].

Following these interaction effects between GSPE consumption and photoperiod, we observed that when proanthocyanidins were consumed at L6 photoperiod, the obesity-driven changes on the gene expression of iWAT were reverted. Thus, while the CAF diet induced the mRNA expression of genes related to adipogenesis (*Pparγ, Cebpα*), lipid transport (*Fabp4*, *Cd36*), lipolysis (*Atgl, Hsl*), and adipokines (*Lep*, *Adipoq*), GSPE was capable of returning the expression of these genes to a healthy situation. In this context, (poly)phenol-induced reduction of lipid turnover has been reported previously in human and animal studies [45,46,47], which partly agrees with our results in the L6 group. However, a significant effect was also observed only under L18, where GSPE consumption upregulated the expression of *Adipoq*. High adiponectin serum concentrations are associated with anti-inflammatory effects that help prevent the metabolic consequences of an obesogenic environment [48]. In this context, a previous study reported that GSPE administration lowered inflammatory levels and increased adiponectin mRNA levels in the mesenteric adipose tissue of obese rats, which agrees with our findings [49]. Importantly, in our study, we could not observe the same effects in animals exposed to L12 and L6, which reinforce the photoperiod-dependent effects of proanthocyanidins on the modulation of gene expression in WAT.

Importantly, we also detected a browning effect in iWAT only in animals consuming proanthocyanidins under short photoperiods. In fact, GSPE was associated with increased thermogenesis in the adipose tissue of obese mice [36], but these results were obtained after supplementing with noticeable higher doses of GSPE. Therefore, in our study, it is possible that the dose of GSPE was too low to fully induce browning in the iWAT. In fact, proanthocyanidins did not seem to promote enhanced BAT activity in our study, as *Ucp1* gene expression was unchanged after both the CAF diet and (poly)phenols consumption. However, other molecules involved in BAT thermogenesis were affected by GSPE in a photoperiod-dependent manner. Thus, proanthocyanidins upregulated the expression of *Pgc1α*, *Cpt1β* and *Pparα* in animals exposed to L12 and L6, while these genes were downregulated in L18 animals. Our results agree with previous findings [27], where GSPE reversed the obesity-induced mitochondrial dysfunction in BAT by increasing *Pgc1α* expression. However, in our study, we demonstrated that these effects are limited to L6 and L12 photoperiods.

In conclusion, our results indicate that the beneficial effects associated with GSPE consumption on diet-induced obese animals are strongly influenced by the photoperiod of exposure in a tissue-specific manner. Particularly, our findings suggest that L18 photoperiod is highly sensitive to GSPE with respect to body weight adaptation and modulation of metabolic genes, especially adiponectin, in the iWAT. Meanwhile, animals under the L12 and L6 photoperiods showed reduced food intake in response to GSPE and enhanced BAT activity. Therefore, our study reinforces the relevance of considering seasonal rhythms when investigating the metabolic effects of (poly)phenols in the organism in order to properly potentiate the metabolic response of the adipose tissue.

## Figures and Tables

**Table 1:** Biometric and circulating parameters.

	P	STD-VH	CAF-VH	CAF-GSPE	*p* Diet ^1^	*p* Diet × P ^1^	*p* GSPE ^1^	*p* GSPE × P ^1^
Body weight (g)	L12	479.8 ± 12.1 ^a^	584.5 ± 11.9 ^b^	555.5 ± 14.2 ^b^	<0.0001	*ns*	*ns*	*ns*
	L18	491.9 ± 12.9 ^a^	598.3 ± 11.4 ^b^	559.1 ± 15.9 ^b^				
	L6	491.4 ± 12.0 ^a^	554.9 ± 19.9 ^b^	554.8 ± 18.5 ^b^				
Body weight gain (g)	L12	96.5 ± 8.9 ^a^	180. ± 10.5 ^b^	156.5 ± 9.7 ^b^	<0.0001	*ns*	0.0403	*ns*
	L18	104.8 ± 9.1 ^a^	197.3 ± 8.2 ^c^	159.4 ± 12.8 ^b^				
	L6	106.1 ± 8.1 ^a^	158.3 ± 14.9 ^b^	155.8 ± 16.1 ^b^				
Food intake (g)	L12	201.3 ± 3.4 ^a^	308.9 ± 14.2 ^c^	256.8 ± 16.4 ^b^	<0.0001	*ns*	0.0015	*ns*
	L18	212.1 ± 6.9 ^a^	301.8 ± 13.4 ^b^	292.1 ± 12.8 ^b^				
	L6	219.3 ± 2.2 ^a^	311.9 ± 7.8 ^c^	264.3 ± 12.7 ^b^				
Fat mass (g)	L12	35.3 ± 3.4 ^a^	83.5 ± 5.3 ^b^	82.2 ± 4.8 ^b^	<0.0001	*ns*	*ns*	*ns*
	L18	46.5 ± 3.5 ^a^	95.1 ± 5.4 ^b^	82.5 ± 6.8 ^b^				
	L6	40.1 ± 3.7 ^a^	74.3 ± 9.4 ^b^	76.1 ± 5.9 ^b^				
iWAT mass (g)	L12	4.1 ± 0.3 ^a^	8.6 ± 0.7 ^b^	9.4 ± 1.1 ^b^	<0.0001	*ns*	*ns*	*ns*
	L18	4.8 ± 0.1 ^a^	9.2 ± 1.3 ^b^	7.1 ± 1.1 ^ab^				
	L6	4.2 ± 0.6 ^a^	8.2 ± 1.5 ^b^	7.2 ± 0.8 ^ab^				
eWAT mass (g)	L12	7.4 ± 0.8 ^a^	18.9 ± 1.1 ^b^	18.1 ± 1.1 ^b^	<0.0001	*ns*	*ns*	*ns*
	L18	10.9 ± 0.5 ^a^	19.6 ± 1.5 ^b^	16.4 ± 1.7 ^b^				
	L6	9.3 ± 0.9 ^a^	16.0 ± 1.7 ^b^	18.6 ± 1.1 ^b^				
BAT mass (g)	L12	0.54 ± 0.05 ^a^	0.96 ± 0.07 ^b^	0.94 ± 0.09 ^b^	<0.0001	*ns*	*ns*	*ns*
	L18	0.49 ± 0.05 ^a^	0.92 ± 0.16 ^ab^	0.99 ± 0.14 ^b^				
	L6	0.64 ± 0.06 ^a^	0.93 ± 0.12 ^ab^	1.01 ± 0.10 ^b^				
Glucose (mmol/L)	L12	5.7 ± 0.3 ^a^	8.1 ± 0.3 ^b^	7.1 ± 0.4 ^ab^	<0.0001	*ns*	*ns*	*ns*
	L18	5.7 ± 0.2 ^a^	7.1 ± 0.2 ^b^	7.6 ± 0.1 ^b^				
	L6	6.3 ± 0.1	7.4 ± 0.4	6.9 ± 0.4				
Cholesterol (mmol/L)	L12	1.23 ± 0.07 ^a^	1.95 ± 0.28 ^b^	1.23 ± 0.09 ^a^	*ns*	*ns*	*ns*	0.0056
	L18	1.28 ± 0.11 ^ab^	1.18 ± 0.10 ^a^	1.57 ± 0.08 ^b^				
	L6	1.20 ± 0.06	1.36 ± 0.19	1.15 ± 0.14				
NEFAs (mmol/L)	L12	0.57 ± 0.06 ^a^	1.17 ± 0.12 ^b^	0.82 ± 0.01 ^a^	0.0003	0.0024	*ns*	0.0005
	L18	0.69 ± 0.07 ^a^	0.64 ± 0.09 ^a^	1.03 ± 0.10 ^b^				
	L6	0.60 ± 0.06 ^a^	0.93 ± 0.10 ^b^	0.68 ± 0.05 ^ab^				
TG (mmol/L)	L12	0.79 ± 0.04 ^a^	2.79 ± 0.36 ^c^	1.82 ± 0.16 ^b^	<0.0001	*ns*	*ns*	*ns*
	L18	0.97 ± 0.13 ^a^	1.88 ± 0.25 ^b^	2.18 ± 0.23 ^b^				
	L6	0.88 ± 0.11 ^a^	2.12 ± 0.39 ^b^	1.49 ± 0.20 ^ab^				

Values are presented as the mean ± SEM of eight animals per group. ^1^ Denotes two-way ANOVA analysis: Diet: diet increasing effect within VH groups; Diet × P: interaction increasing effect between diet and photoperiod within VH groups; GSPE: GSPE consumption decreasing effect within CAF groups; GSPE × P: interaction decreasing effect between GSPE consumption and photoperiod within CAF groups; *ns:* no significant effects. Different letters denote significant differences within each photoperiod group (assessed with one-way ANOVA followed by Tukey’s *post hoc* test, *p* < 0.05). BAT: brown adipose tissue; CAF: cafeteria diet; eWAT: epididymal white adipose tissue; GSPE: grape-seed proanthocyanidins extract; iWAT: inguinal white adipose tissue; NEFAs: non-esterified fatty acids; P: photoperiod; STD: standard diet; TG: triglycerides; VH: vehicle

## Data Availability

The data presented in this study are available on request from the corresponding author. The data are not publicly available due to a lack of a platform to publish them.

## Supplementary Materials

The following supporting information can be downloaded at: https://www.mdpi.com/article/10.3390/nu15041037/s1, Figure S1: Experimental design used in this study. Figure S2: Representative images of iWAT histology; Figure S3: Effect of grape seed proanthocyanidin extract (GSPE) consumption at different photoperiods, 12 h of light:12 h of darkness (L12); 18 h of light:6 h of darkness (L18); 6 h of light:18 h of darkness (L6), on the gene expression of *Acacα* and *Fasn* in inguinal white adipose tissue (iWAT) of diet-induced obese rats; Figure S4: Representative images of eWAT histology; Figure S5: Effect of grape seed proanthocyanidin extract (GSPE) consumption at different photoperiods, 12 h of light:12 h of darkness (L12); 18 h of light:6 h of darkness (L18); 6 h of light:18 h of darkness (L6), on the expression of lipogenesis and inflammation-related genes in epididymal white adipose tissue (eWAT) of diet-induced obese rats; Figure S6. Effect of grape seed proanthocyanidin extract (GSPE) consumption at different photoperiods, 12 h of light:12 h of darkness (L12); 18 h of light:6 h of darkness (L18); 6 h of light:18 h of darkness (L6), on the expression of key metabolic genes in brown adipose tissue (BAT) of diet-induced obese rats; Table S1: Phenolic Composition of GSPE; Table S2: Primers for the Q-PCR analysis.
