# Supplementary material for: Grape-Seed Proanthocyanidins Modulate Adipose Tissue Adaptations to Obesity in a Photoperiod-Dependent Manner in Fischer 344 Rats
Source: Nutrients. 2023 Feb 19;15(4):1037. doi: 10.3390/nu15041037 (PMC9967183; doi:10.3390/nu15041037)
Supplement: Supplementary file 1 [file nutrients-15-01037-s001.zip › nutrients-2236502-supplementary.pdf]

Table S1. Phenolic Composition of GSPE

Phenolic Compound	Concentration (μmol/g)
Catechin	58
Dimeric procyanidins	250
Epicatechin	52
Epigallocatechin	5.50
Epicatechin gallate	89
Epigallocatechin gallate	1.40
Hexameric procyanidins	0.38
Pentameric procyanidins	0.73
Tetrameric procyanidins	8.8
Trimericprocyanidins	1568

Table S2. Primers for the Q-PCR analysis.

	Forward (5'...3')	Reverse (5'...3')
Ucp1	GGTACCCACATCAGGCAACA	TCTGCTAGGCAGGCAGAAAC
Prdm16	GTTCTGCGTGGATGCCAATC	TGGCGAGGTTTTGGTCATCA
Cpt1β	GCAAACTGGACCGAGAAGAG	CCTTGAAGAAGCGACCTTTG
Cd36	CAGTGCAGAAACAGTGGTTGTCT	TGACATTTGCAGGTCCATCTATG
Ppia	CTTCGAGCTGTTTGCAGACAA	AAGTCACCACCCTGGCACATG
Pparγ	AGGGCGATCTTGACAGGAAA	CGAAACTGGCACCCTTGAAA
C/ebpα	TGTACTGTATGTCGCCAGCC	TGGTTTAGCATAGACGCGCA
Acacα	GCGGCTCTGGAGGTATATGT	TCTGTTTAGCGTGGGGATGT
Fasn	TAAGCGGTCTGGAAAGCTGA	CACCAGTGTTTGTTCCTCGG
Atgl	GAAGACCCTGCCTGCTGATT	CACATAGCGCACCCCTTGAA
Hsl	AGTTCCCTCTTTACGGGTGG	GCTTGGGGTCAGAGGTTAGT
Pparα	CGGCGTTGAAAACAAGGAGG	TTGGGTTCCATGATGTCGCA
Fabp4	GAAAGAAGTGGGAGTTGGCT	TACTCTCTGACCGGATGACG
Dio2	TTATGGGGTAGCCTTTGAACG	CCAGCCAACTTCGGACTT
Pgc1a	AGAGTCACCAAATGACCCCAAG	TTGGCTTTATGAGGAGGAGTCG
Lep	ATTTCACACACGCAGTCGGTAT	CCCGGGAATGAAGTCCAAA
Adipoq	GTTCCAGGACTCAGGATGCT	CGTCTCCCTTCTCTCCCTTC
Il6	ATATGTTCTCAGGGAGATCTTGGAA	TGCATCATCGCTGTTCATACAA
Tnfα	CGTCAGCCGATTTGCCATTTC	TGGGCTCATACCAGGGCTTGAG

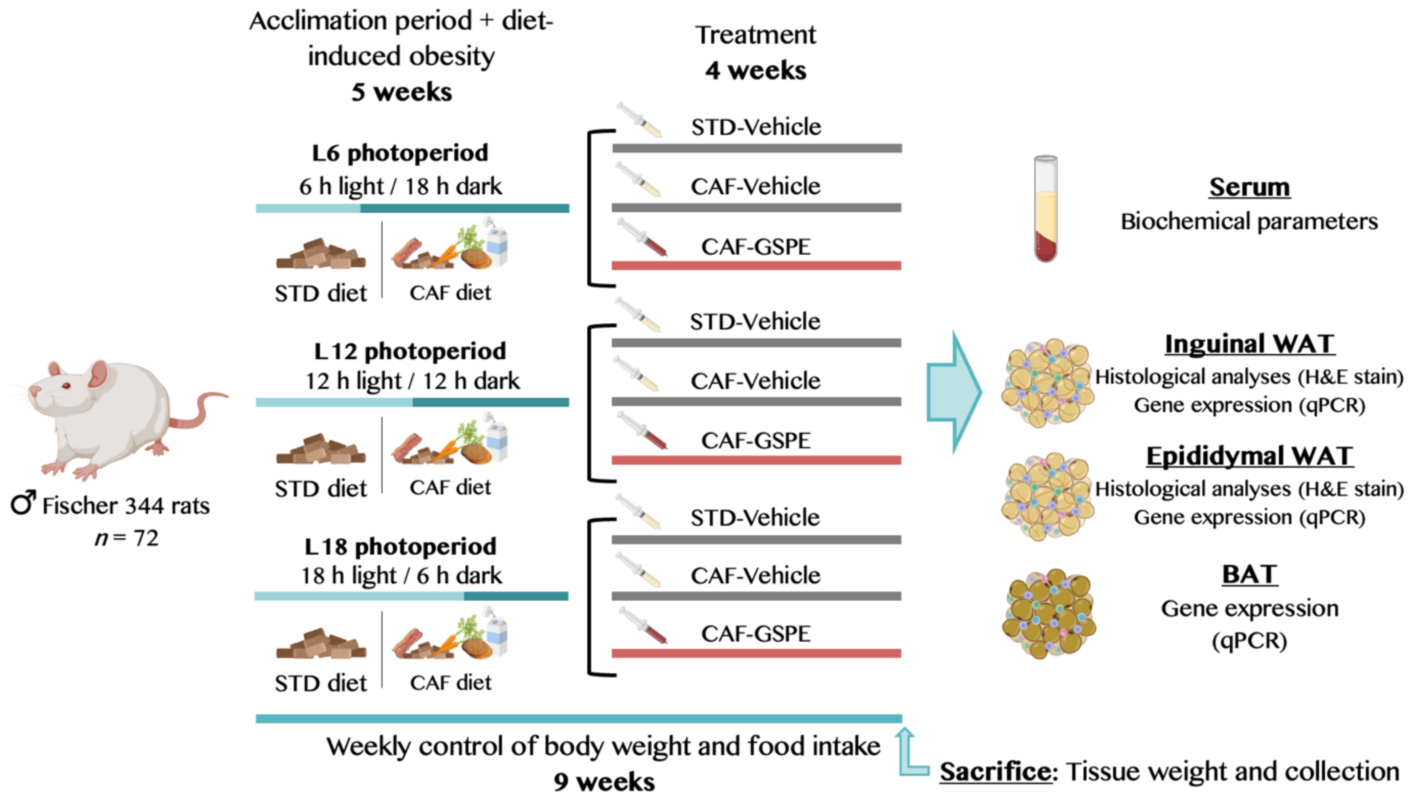

Figure S1. Experimental design used in this study.

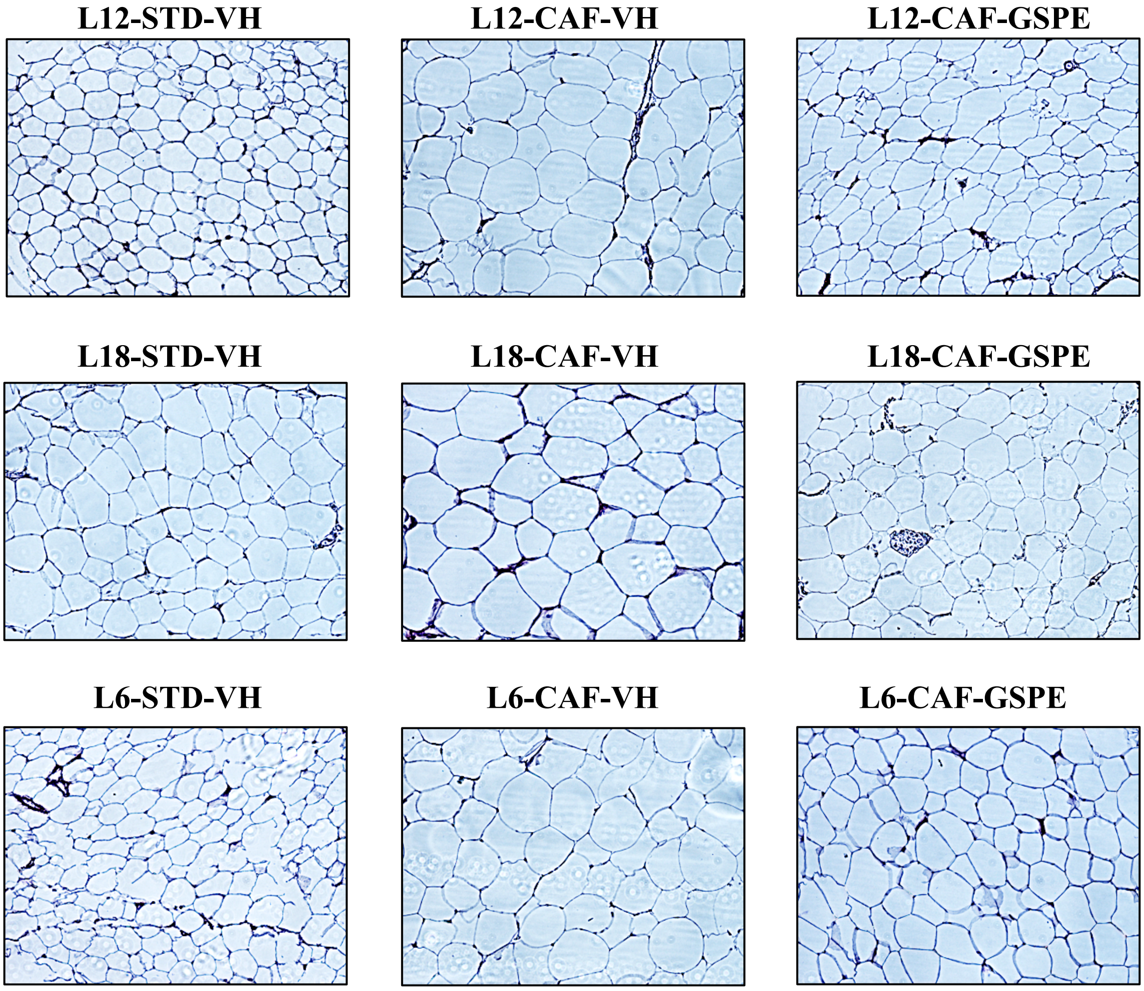

Figure S2. Representative images of iWAT histology. Histological study of iWAT morphology of Fischer 344 rats fed with standard diet (STD) or cafeteria diet (CAF) for 9 weeks and treated with GSPE (25 mg/kg body weight) or vehicle (VH) the last 4 weeks of the study; STD-VH group; CAF-VH group; CAF-GSPE group, under different photoperiods; L12, L18 or L6.

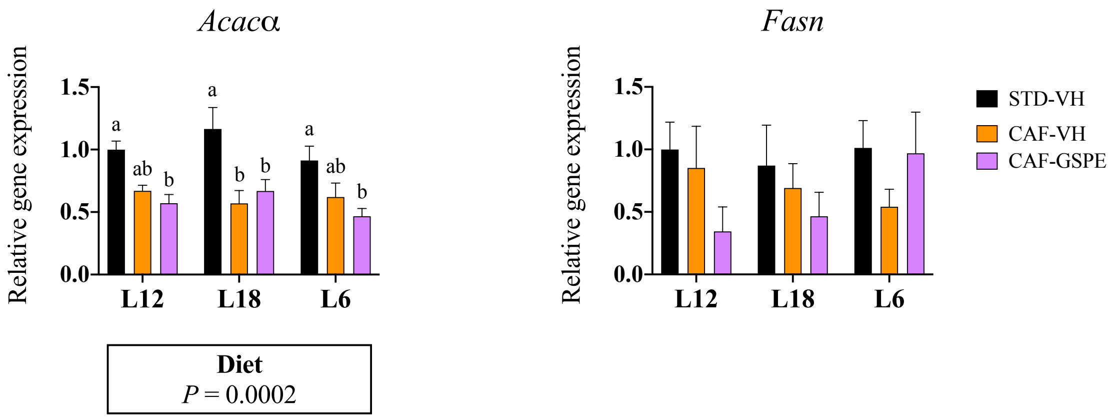

Figure S3. Effect of grape seed proanthocyanidin extract (GSPE) consumption at different photoperiods, 12h of light:12h of darkness (L12); 18h of light:6h of darkness (L18); 6h of light:18h of darkness (L6) on the gene expression of *Acacα* and *Fasn* in inguinal white adipose tissue (iWAT) of diet-induced obese rats. Fisher 344 rats were fed standard diet (STD) or cafeteria diet (CAF) for 9 weeks and treated with GSPE (25 mg/kg body weight) or vehicle (VH) the last 4 weeks of the study; STD-VH group; CAF-VH group; CAF-GSPE group, under different photoperiods; L12, L18 or L6. The gene expression was measured by qPCR and normalized by *Ppia*. The relative expression (presented as fold-change) of CAF-VH and CAF-GSPE was normalized to the corresponding STD-VH control group (L12 photoperiod). Significant differences were assessed through two-way ANOVA analysis ($P < 0.05$). **Diet:** diet effect within VH groups. Different letters denote significant differences within each photoperiod group (assessed with two-way ANOVA followed by Tukey's *post hoc* test, $p < 0.05$).

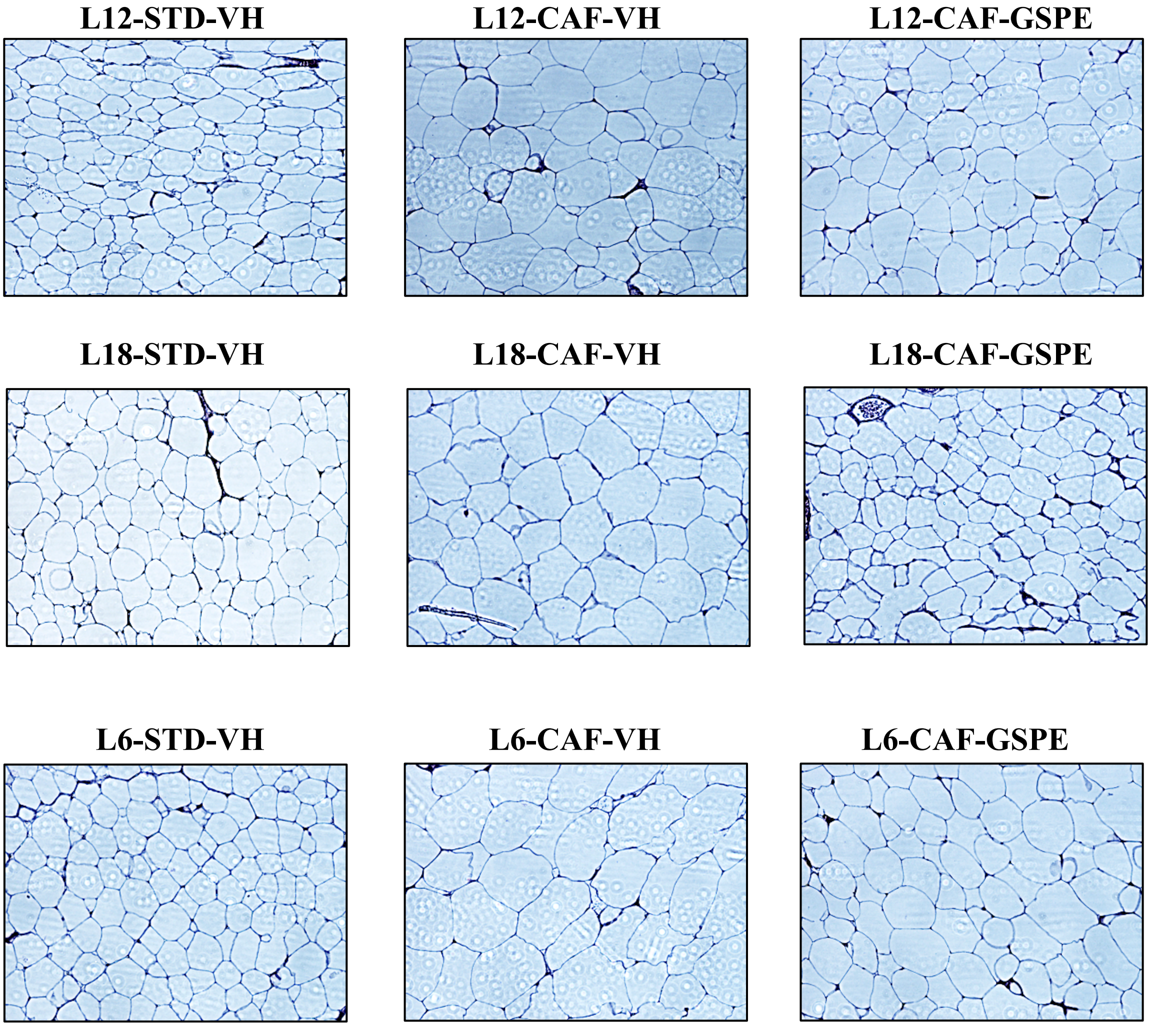

Figure S4. Representative images of eWAT histology. Histological study of eWAT morphology of Fischer 344 rats fed with standard diet (STD) or cafeteria diet (CAF) for 9 weeks and treated with GSPE (25 mg/kg body weight) or vehicle (VH) the last 4 weeks of the study; STD-VH group; CAF-VH group; CAF-GSPE group, under different photoperiods; L12, L18 or L6.

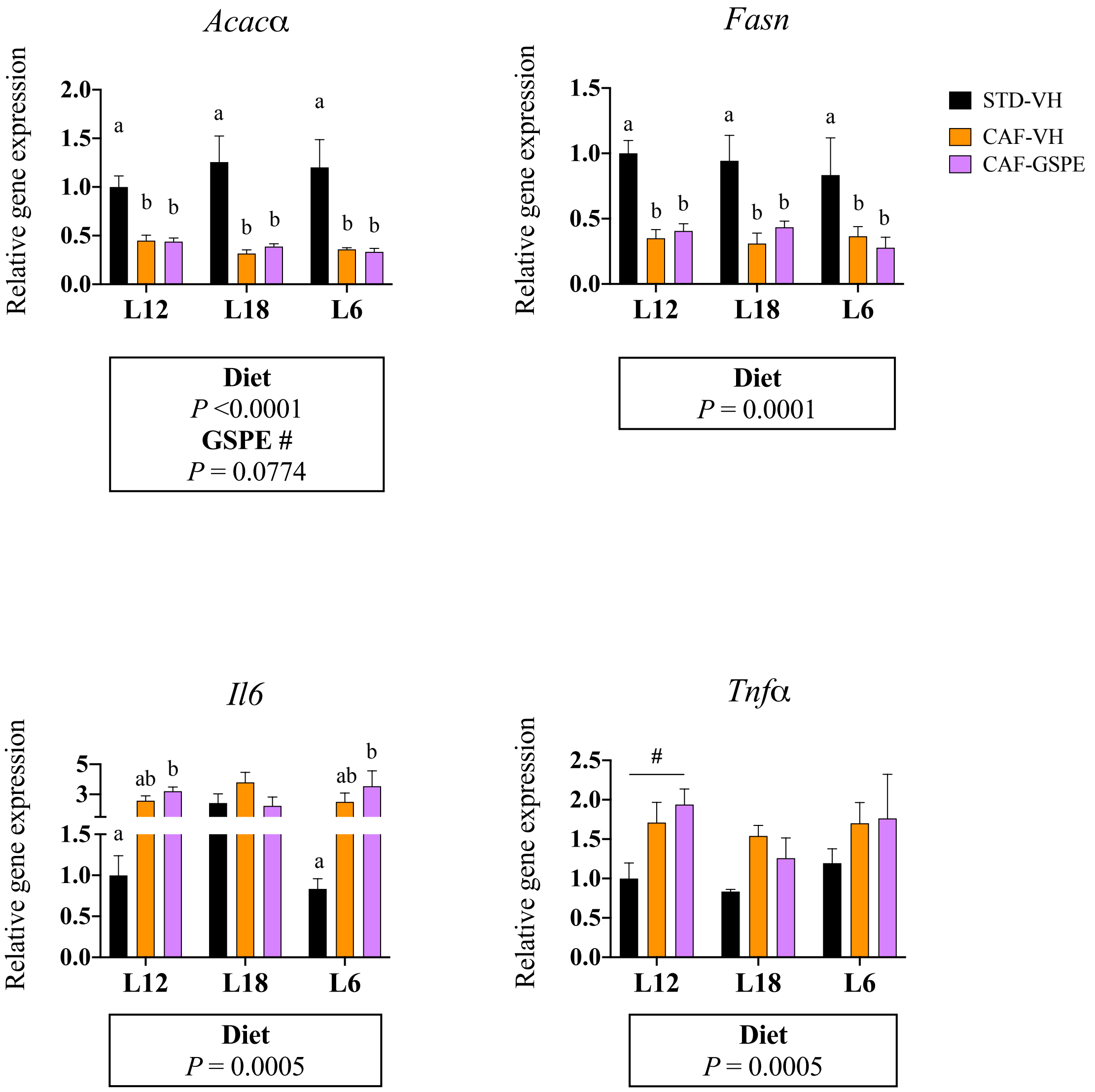

Figure S5. Effect of grape seed proanthocyanidin extract (GSPE) consumption at different photoperiods, 12h of light:12h of darkness (L12); 18h of light:6h of darkness (L18); 6h of light:18h of darkness (L6) on the expression of lipogenesis and inflammation-related genes in epididymal white adipose tissue (eWAT) of diet-induced obese rats. Fisher 344 rats were fed standard diet (STD) or cafeteria diet (CAF) for 9 weeks and treated with GSPE (25 mg/kg body weight) or vehicle (VH) the last 4 weeks of the study; STD-VH group; CAF-VH group; CAF-GSPE group, under different photoperiods; L12, L18 or L6. The gene expression was measured by qPCR and normalized by *Ppia*. The relative expression (presented as fold-change) of CAF-VH and CAF-GSPE was normalized to the corresponding STD-VH control group (L12 photoperiod). Significant differences were assessed through two-way ANOVA analysis ($P < 0.05$). **Diet:** diet effect within VH groups; **GSPE**: GSPE effect within CAF groups. Different letters denote significant differences within each photoperiod group (assessed with two-way ANOVA followed by Tukey's *post hoc* test, $p < 0.05$; #: $p < 0.1$).

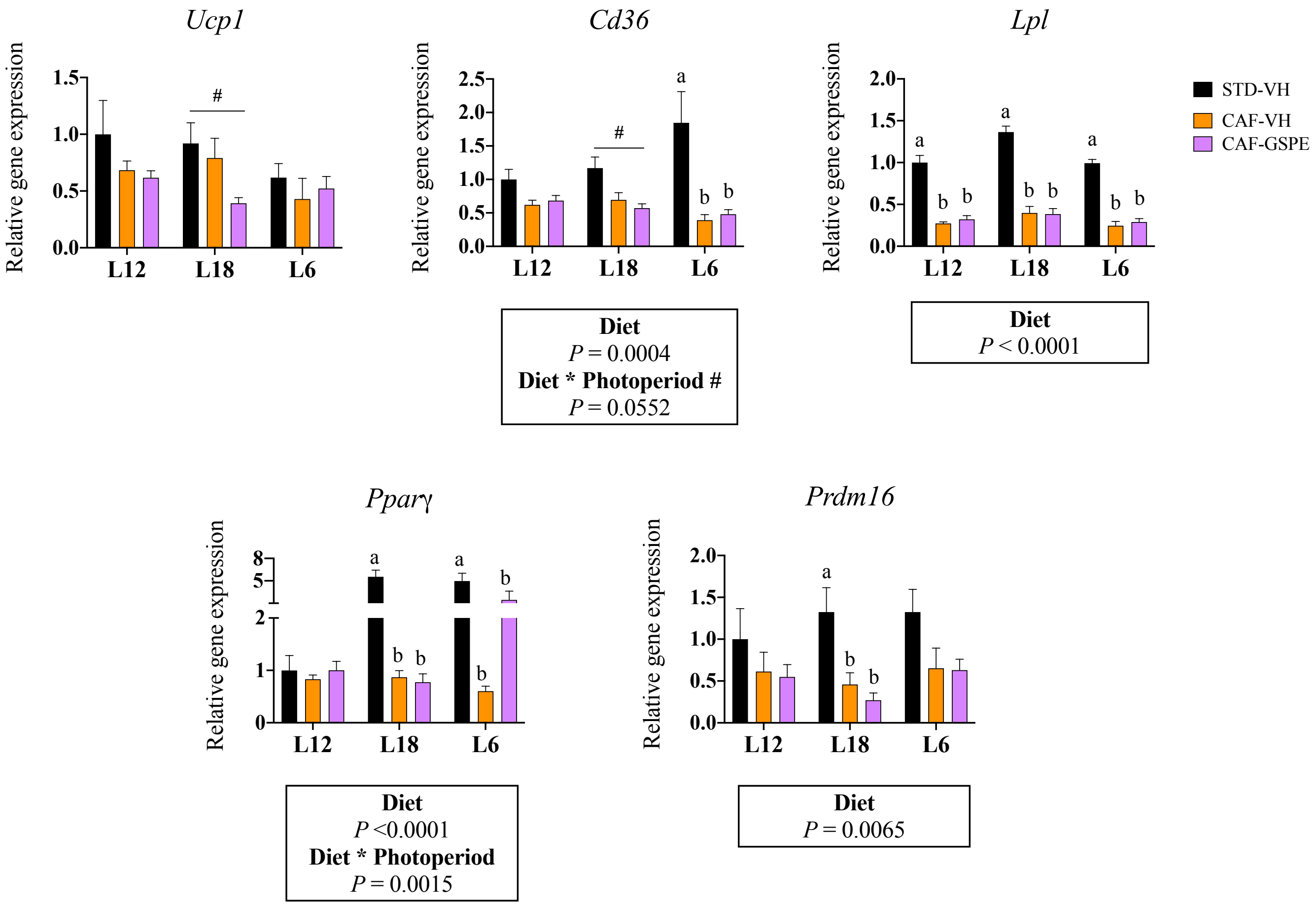

Figure S6. Effect of grape seed proanthocyanidin extract (GSPE) consumption at different photoperiods, 12h of light:12h of darkness (L12); 18h of light:6h of darkness (L18); 6h of light:18h of darkness (L6) on the expression of key metabolic genes in brown adipose tissue (BAT) of diet-induced obese rats. Fisher 344 rats were fed standard diet (STD) or cafeteria diet (CAF) for 9 weeks and treated with GSPE (25 mg/kg body weight) or vehicle (VH) the last 4 weeks of the study; STD-VH group; CAF-VH group; CAF-GSPE group, under different photoperiods; L12, L18 or L6. The gene expression was measured by qPCR and normalized by *Ppia*. The relative expression (presented as fold-change) of CAF-VH and CAF-GSPE was normalized to the corresponding STD-VH control group (L12 photoperiod). Significant differences were assessed through two-way ANOVA analysis ($P < 0.05$). **Diet:** diet effect within VH groups; **Diet * Photoperiod**: interaction effect between CAF diet and photoperiod within VH groups. Different letters denote significant differences within each photoperiod group (assessed with two-way ANOVA followed by Tukey's *post hoc* test, $p < 0.05$; #: $p < 0.1$).
